# Comparison of a Bayesian Network with a Logistic Regression Model to Forecast IgA Nephropathy

**DOI:** 10.1155/2013/686150

**Published:** 2013-11-17

**Authors:** Michel Ducher, Emilie Kalbacher, François Combarnous, Jérome Finaz de Vilaine, Brigitte McGregor, Denis Fouque, Jean Pierre Fauvel

**Affiliations:** ^1^Département de Néphrologie et Hypertension, Hospices Civils de Lyon, Hôpital Edouard Herriot, 69437 Lyon, France; ^2^Clinique du Tonkin, 26 rue du Tonkin, 69100 Villeurbanne, France

## Abstract

Models are increasingly used in clinical practice to improve the accuracy of diagnosis. The aim of our work was to compare a Bayesian network to logistic regression to forecast IgA nephropathy (IgAN) from simple clinical and biological criteria. Retrospectively, we pooled the results of all biopsies (*n* = 155) performed by nephrologists in a specialist clinical facility between 2002 and 2009. Two groups were constituted at random. The first subgroup was used to determine the parameters of the models adjusted to data by logistic regression or Bayesian network, and the second was used to compare the performances of the models using receiver operating characteristics (ROC) curves. IgAN was found (on pathology) in 44 patients. Areas under the ROC curves provided by both methods were highly significant but not different from each other. Based on the highest Youden indices, sensitivity reached (100% versus 67%) and specificity (73% versus 95%) using the Bayesian network and logistic regression, respectively. A Bayesian network is at least as efficient as logistic regression to estimate the probability of a patient suffering IgAN, using simple clinical and biological data obtained during consultation.

## 1. Introduction

Modeling techniques are increasingly used in medicine to help make medical diagnoses (clinical prediction rules) and predict treatment efficacy. Such an approach has never been used in nephrology to forecast IgA nephropathy which is the most frequent form of glomerulonephritis [1]. Its annual incidence is of 5 to 12 new cases per million inhabitants in the United States [2] and 15 to 40 in Europe [3–5]. Depending on studies, it accounts for 7 to 52% of primary glomerular diseases [6–8]. Amongst all forms of primary glomerular disease in Europe today, IgAN is the commonest cause of end-stage renal failure [9–11]. Diagnosis of IgA nephropathy relies on microscopic examination of renal biopsies and is confirmed by immunocytochemical techniques. Biopsy, which is necessary for confirming the diagnosis, may lead to severe complications. Thanks to the development of predictive models, IgA nephropathy could be, in the future, diagnosed more quickly and patients could be treated earlier. Logistic regressions are commonly used to forecast the probability of diseases. Nonparametric approaches such as Bayesian networks used in computer science and engineering could be an effective alternative in medicine. Bayesian networks, based on demographic, clinical, biological, and radiological parameters, have been used to develop diagnostic and prognostic tools in a variety of research fields to support medical decision-making [12–16]. The aim of our study was to check if IgAN could be predicted using simple clinical and biological data. To our knowledge, such a methodology has never been tested in nephrology. For this purpose, we used retrospective data and tested the best way to adjust the prediction models to our data. Thus, we compared performances of a Bayesian network with those of logistic regression to forecast IgA nephropathy (IgAN) from simple clinical and biological criteria collected during a medical consultation.

## 2. Material and Methods

### 2.1. Patients

In this retrospective study, we included all patients who underwent a first renal biopsy at the Tonkin Clinic between January 1, 2002 and December 31, 2009, irrespective of the reasons. Clinical and biological information was obtained before renal biopsy from the patients' files in the Nephrology Department. The inclusion criteria were *patients aged over 18 years, first biopsy performed between 2002 and 2009, and biopsy of native kidney.*

Biopsy specimens were analyzed by the same operator in the same laboratory by means of optical microscopy followed by immunofluorescence using polyclonal antibodies to IgG, IgM, IgA, C3, C1q, kappa, and lambda free light chains.

Routine clinical and demographic data collected were:

- *demographic*: gender and age at biopsy;
- *clinical*: weight, personal history of microscopic and/or macroscopic hematuria, family history of hematuria and/or nephropathy, eye or ear disease, personal history of diabetes, and/or hypertension, and history of lupus nephritis;
- *biological*: serum creatinine to calculate renal clearance according to the modification of the diet in renal disease (MDRD) formula [17], serum immunoglobulin A (IgA), and urinary data: presence and quantification of hematuria, 24 h proteinuria.

Natures of variables were continuous (age, eGFR, and proteinuria), categorical (serum IgA), or dichotomous (gender, history of hypertension, microhematuria, gross hematuria, family history of hematuria, and history of diabetes) for the logistic regression (Table 1). To enter the Bayesian model, variables were categorized as defined in Table 1. Of the biological tests, only the measurement of serum IgA level was not performed routinely. There were three possible data entries for IgA status: high serum IgA, normal serum IgA or “not done.” In this retrospective study, data management and analysis complied with the French law and the Declaration of Helsinki. A local ethics committee approved this retrospective study and data were recorded anonymously.

### 2.2. Construction of Models

Biopsy reports (including immunofluorescence results) were used as the “gold standard” for predicting models and ROC curve analysis. IgAN represented the end-point (clearly defined on pathology) with a status of 1 versus non-IgAN with a status of 0 (other different renal diseases). Stepwise multiple logistic regression was performed to assess the variables significantly linked to the probability of IgAN. We used a stepwise multiple logistic regression model because it was well suited to our main objective and to our data. Logistic regression provides individual probability of having a disease. Logistic regression does not assume hypotheses on variable distribution (homogeneity of variances). Stepwise methodology avoids collinearity between variables. A *P* value <0.05 was considered significant.

Bayesian networks belong to the family of graphical models. The network structure can be described as follows: each node in the graph represents a variable, while the edges between the nodes represent probabilistic dependencies among the corresponding variables.

### 2.3. Statistical Analysis and Validation of the Models

Qualitative variables were described by number and frequency and quantitative variables by mean ± SEM. Means were compared using a Student's *t*-test and distributions by using a Chi^2^ test. 

Seventy-five (75) patients selected randomly from the cohort formed the “learning sample.” The remaining patients (*n* = 74) formed the validation sample. The models were built using the “learning sample.” Performances of models were then tested using ROC curve analysis on the validation sample. ROC curve represents sensitivities and (1-specificities) according to different cut-offs. Area under the curve (AUC, C statistics) was used to evaluate the “overall diagnostic accuracy” of the test in relation to biopsy results. We compared areas under the ROC curves to highlight a statistical difference between performances of the two models. Software used for statistical tests (including stepwise regression analysis and ROC analysis) was Medcalc 11.5.1.0 version, and Netica (Norsys Software Corp.) was used for the Bayesian model.

## 3. Results

From January 1, 2002 to December 31, 2009, 155 patients underwent first renal biopsy. Six of the histological specimens did not allow for complete analysis by optical microscopy and/or immunofluorescence. Therefore, these histological reports were not included in our analysis. For the 149 patients whose biopsy specimens were analyzable, most histological reports indicated IgAN (30%), focal segmental glomerulosclerosis (15%), minimal change glomerulonephritis (11%), vascular nephropathy (9%), interstitial nephritis (9%), membranous glomerulonephritis (6%), lupus nephritis (2%), diabetes nephropathy (4%), and other diagnoses (14%). Of the 44 IgAN, 42 patients had Berger's disease and two had rheumatoid purpura. Mean age was 52 years, predominantly male (71%).

The general characteristics of the 149 patients are shown in Table 1. Probability distribution of modalities for all the variables expressed as percentages for the learning sample represents the belief of the network (Table 2). The characteristics of patients randomized to constitute the learning sample were comparable to those of patients used to validate the model (Table 2).

Using the stepwise multivariate logistic regression, microscopic hematuria and serum IgA were the only variables statistically linked to IgAN (*P* < 0.0001 and *P* = 0.01, resp.). The equation of the logistic regression was Logit (*P*(IgAN)) = 3.01 (microscopic hematuria) + 2.03 (high serum IgA) − 2.60.

All the variables could be included in the Bayesian model. Quantifications of the linkage between nodes (Kullback-Leibler distance) are given in Table 3. The Kullback-Leibler distance is used to assess the degree of dependence between two variables of the network. Microscopic hematuria was the variable most related to IgAN with the Bayesian model.

For each patient in the validation sample, the models were used to compute the probability of IgAN. 

Areas under the ROC curves were 0.83 and 0.75 for the Bayesian model and logistic regression, respectively. Areas under the ROC curves were not statistically different between the two models.

**Figure 1:**
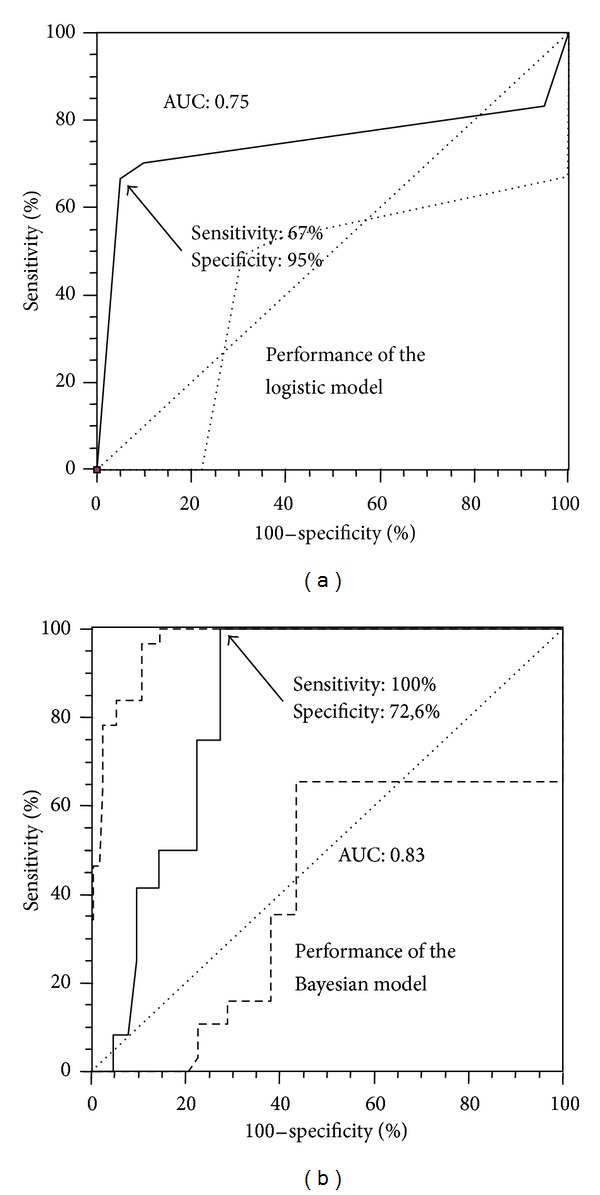
Receiver operating characteristic curves used to assess the predictive values of the two models to diagnose IgAN in the validation sample of 74 patients. AUC means area under the curve.

Based on the highest Youden indices, sensitivity reached (100% versus 67%) and specificity reached (73% versus 95%) using the Bayesian network and the logistic regression, respectively, as shown by the ROC curves (Figure 1). Positive predictive values (PPV) were 64% and 47% and negative predictive values (NPV) were 98% and 95% for the Bayesian network and logistic regression, respectively.

## 4. Discussion

In this study we demonstrated that a Bayesian network using simple clinical and biological parameters collected during consultation was at least as efficient as logistic regression for estimating the probability of a patient having IgAN. Using both methods, the areas under the ROC curves were highly statistically significant. However, areas under the ROC curves were not statistically different between methods.

IgAN was chosen as a target since it is the most prevalent glomerulopathy. The mean age of patients requiring biopsy is increasing constantly [6], which explains why the mean age in our sample is slightly higher than that in earlier studies [3]. In our sample, renal function at biopsy was lower (63 mL/min/1/1.73 m²) than that usually reported. The proportion of hypertensive patients was similar to that which is in other reports in the literature (44%) [18–20]. The sex ratio was comparable with that in other studies. Like others, we found no significant age difference between men and women with IgAN (40.0 ± 3.8 versus 42.6 ± 2.9 years, resp.). The proportion of patients with IgAN was similar to that generally reported. We noted a higher number of vascular nephropathy and focal segmental glomerulosclerosis cases, probably due to the higher percentage of elderly, hypertensive patients [18–20].

Logistic regression models are often used to predict the likelihood of disease. They have been used to build clinical prediction rules to help physicians in decision-making [21]. However, multivariate regression models have some limitations [22]. Regression models are mainly influenced by the sample size and cannot manage missing data. Unfortunately, in clinical practice missing data is a common occurrence and clinical prediction based on regression models cannot provide probability of disease in this event. Regression analyses adjust models (coefficients and/or variables) to data. The parameters of clinical prediction rules obtained by regression are frozen and may therefore be unsuitable for patients coming from different clinical centers [21, 23].

Bayesian models overcome the limitations of regression modelling. Interestingly, Bayesian networks can simultaneously process quantitative data (creatinine, proteinuria) and qualitative data (gender). The advantage of a Bayesian model is that there is no *a priori* hypothesis about the nature of the modeled relationships. It is of interest to note that the two descriptors used for logistic regression also have a higher statistical link for Bayesian network. A Bayesian network using all ten clinical and biological descriptors to give a prediction is more robust than logistic regression. Furthermore, the Bayesian model tolerates missing data and manages any inconsistencies, either in the learning database or during application. For example, given a missing datum, the software uses a computed value, that is, the calculated probability of having this missing datum according to other variables which depend on it. Another advantage is the possibility of enriching network knowledge from new cases to calculate further probabilities. This continual apprenticeship makes it possible to refine the predictive quality of the model and to adapt medical practices in each center accordingly. Medical and recruitment practices may vary among centers. Thus, with a shared database, each center can enter its own data and enrich the overall software apprenticeship. Probability values will be closer to the real situation in each center where practices may change over time.

Applied to real data, Bayesian networks have already proven their ability to outperform logistic regression [14, 24] in terms of diagnosis prediction. Our results are in accordance with this assertion. When screening for diseases, sensitivity is paramount. Reaching 100%, the sensitivity of the Bayesian network was higher compared to the sensitivity obtained using logistic regression (67%). For a pragmatic approach, positive (PPV) and negative predictive values (NPV) are more suitable to check the ability of a test to establish or to eliminate a diagnosis. The Bayesian model which has higher PPV and similar NPV appears to be better suited to clinical practice than does logistic regression.

We present a pilot study which aimed to evaluate Bayesian network ability to diagnose IgAN. To date, the treatment of IgAN relies on its histological confirmation and two biological parameters [25]. Furthermore, histology provides prognostic information on renal function decline [26, 27]. The reliability of the diagnosis provided by Bayesian modeling is not sufficient to avoid performing renal biopsies to prove IgAN and initiate a treatment. However, renal biopsy can be risky, although risk is minimized in adequately trained teams. Clinical prediction rules based on Bayesian modeling are useful to justify the need for renal biopsy by analyzing complex information and standardizing the medical process. 

In conclusion, a Bayesian network is at least as efficient as logistic regression for estimating the probability of a patient suffering IgAN, using simple clinical and biological data obtained during consultation. Thus, our proposed model should be regarded as a simple and helpful decision-making tool in the field of nephrology. An external prospective, multicentre validation study is required before using it in clinical practice.

## Figures and Tables

**Table 1:** Characteristics of 149 patients with analyzable renal biopsy specimens between January 2002 and December 2009.

Age (years)	Mean ± SEM	48.2 + 2.01
	Age < 40	36%
	40 ≤ age < 60	34%
	Age ≥ 60	30%
Male		64%
History of hypertension		44%
Microhematuria		45%
Gross hematuria		17%
Family history of hematuria		3%
History of diabetes		11%
Mean eGFR (MDRD mL/min/1.73 m²)	Mean ± SEM	63 + 3.4
Stage of renal failure	eGFR ≤ 29 mL/min/1.73 m²	15%
	30 ≤ eGFR ≤ 59 mL/min/1.73 m²	31%
	60 mL/min/1.73 m² ≤ eGFR	54%
Serum Ig A	Increased > 3.6 g/L	18%
	Normal ≤ 3.6 g/L	34%
	Not performed	48%
Proteinuria (g/24 h)		3.44 + 0.43
	Proteinuria < 0.3 g/24 h	12%
	0.3 g/24 h ≤ proteinuria < 1 g/24 h	20%
	1 g/24 h ≤ proteinuria < 3 g/24 h	34%
	Proteinuria ≥ 3 g/24 h	34%

**Table 2:** Characteristics of patients in the learning versus validation sample.

Group		Learning (*N* = 75)	Validation (*N* = 74)	*P *
Age (years)	Mean ± SEM	48.2 ± 2.06	48.2 ± 2.04	NS
	Age < 40 years	28 (37%)	25 (34%)	NS
	40 ≤ age < 60 years	26 (35%)	25 (34%)	
	Age ≥ 60 years	21 (28%)	24 (32%)	
Male gender		44 (59%)	50 (68%)	NS
History of hypertension		31 (41%)	35 (47%)	NS
Microhematuria		32 (43%)	34 (46%)	NS
Macrohematuria		12 (16%)	13 (18%)	NS
Family history of hematuria		3 (4%)	1 (1%)	NS
History of diabetes		5 (7%)	12 (16%)	NS
Mean GFR (MDRD mL/min/1.73 m²)	Mean ± SEM	62.1 ± 3.5	63.3 ± 3.5	NS
Stage of renal failure	eGFR ≤ 29 mL/min/1.73 m²	11 (15%)	11 (15%)	NS
	30 ≤ eGFR ≤ 59 mL/min/1.73 m²	25 (33%)	21 (28%)	
	60 mL/min/1.73 m² ≤ eGFR	39 (52%)	42 (57%)	
Serum Ig A	Increased	14 (19%)	13 (18%)	NS
	Normal	25 (33%)	26 (35%)	
	Not performed	36 (48%)	35 (47%)	
Proteinuria (g/24 h)	Mean ± SEM	3.43 ± 0.58	3.45 ± 0.65	NS
	Proteinuria < 0.3 g/24 h	8 (10%)	10 (14%)	NS
	0.3 g/24 h ≤ proteinuria < 1 g/24 h	17 (23%)	13 (18%)	NS
	1 g/24 h ≤ proteinuria < 3 g/24 h	26 (35%)	24 (32%)	
	Proteinuria ≥ 3 g/24 h	24 (32%)	27 (36%)	
Number of IgAN		26 (35%)	18 (24%)	NS

**Table 3:** Dependence between variable to predict (IgAN) and predictors expressed as Kullback-Leibler divergence.

Microhematuria	0.29
Gross hematuria	0.14
Serum Ig A	0.07
Proteinuria	0.06
History of diabetes	0.05
Age	0.04
GFR	0.03
History of hypertension	0.02
Family history of hematuria	0.01
Gender	0.001

## References

[1] D’Amico G (1987). The commonest glomerulonephritis in the world: IgA nephropathy. *Quarterly Journal of Medicine*.

[2] Wyatt RJ, Julian BA, Baehler RW (1998). Epidemiology of IgA nephropathy in central and eastern Kentucky for the period 1975 through 1994. Central Kentucky Region of the Southeastern United States IgA Nephropathy DATABANK Project. *Journal of the American Society of Nephrology*.

[3] Simon P, Ramée M-P, Autuly V (1994). Epidemiology of primary glomerular diseases in a French region. Variations according to period and age. *Kidney International*.

[4] Rambausek M, Rauterberg EW, Waldherr R, Demaine A, Krupp G, Ritz E (1987). Evolution of IgA glomerulonephritis: relation to morphology, immunogenetics, and BP. *Seminars in Nephrology*.

[5] Tiebosch ATMG, Wolters J, Frederik PFM (1987). Epidemiology of idiopathic glomerular disease: a prospective study. *Kidney International*.

[6] Stratta P, Segoloni GP, Canavese C (1996). Incidence of biopsy-proven primary glomerulonephritis in an Italian province. *American Journal of Kidney Diseases*.

[7] Levy M, Berger J (1988). Worldwide perspective of IgA nephropathy. *American Journal of Kidney Diseases*.

[8] Li L-S, Liu Z-H (2004). Epidemiologic data of renal diseases from a single unit in China: analysis based on 13,519 renal biopsies. *Kidney International*.

[9] Rychlík I, Jančová E, Tesař V (2004). The Czech registry of renal biopsies. Occurrence of renal diseases in the years 1994–2000. *Nephrology Dialysis Transplantation*.

[10] Valderrábano F, Berthoux FC, Jones EHP, Mehls O (1996). Report on management of renal failure in Europe, XXV, 1994 end stage renal disease and dialysis report. *Nephrology Dialysis Transplantation*.

[11] Kessler M, Ayav C, Erpelding M-L, Couchoud C (2012). Trends in characteristics of ESRD patients at initiation of dialysis therapy. *Nephrologie et Therapeutique*.

[12] Lalande L, Bourguignon L, Carlier C, Ducher M (2013). Bayesian networks: a new method for the modeling of bibliographic knowledge: application to fall risk assessment in geriatric patients. *Medical and Biological Engineering and Computing*.

[13] Zhao D, Weng C (2011). Combining PubMed knowledge and EHR data to develop a weighted bayesian network for pancreatic cancer prediction. *Journal of Biomedical Informatics*.

[14] Sakai S, Kobayashi K, Nakamura J, Toyabe S, Akazawa K (2007). Accuracy in the diagnostic prediction of acute appendicitis based on the Bayesian network model. *Methods of Information in Medicine*.

[15] Burnside ES, Davis J, Chhatwal J (2009). Probabilistic computer model developed from clinical data in national mammography database format to classify mammographic findings. *Radiology*.

[16] Forsberg JA, Eberhardt J, Boland PJ, Wedin R, Healey JH (2011). Estimating survival in patients with operable skeletal metastases: an application of a Bayesian belief network. *PLoS ONE*.

[17] Levey AS, Coresh J, Greene T (2006). Using standardized serum creatinine values in the modification of diet in renal disease study equation for estimating glomerular filtration rate. *Annals of Internal Medicine*.

[18] Manno C, Strippoli GFM, D’Altri C, Torres D, Rossini M, Schena FP (2007). A novel simpler histological classification for renal survival in IgA nephropathy: a retrospective study. *American Journal of Kidney Diseases*.

[19] Li PKT, Ho KKL, Szeto CC, Yu L, Lai FM-M (2002). Prognostic indicators of IgA nephropathy in the Chinese—clinical and pathological perspectives. *Nephrology Dialysis Transplantation*.

[20] Berthoux FC, Mohey H, Afiani A (2008). Natural history of primary IgA nephropathy. *Seminars in Nephrology*.

[21] Krijnen P, Van Jaarsveld BC, Steyerberg EW, Man In ’T Veld AJ, Schalekamp MADH, Habbema JDF (1998). A clinical prediction rule for renal artery stenosis. *Annals of Internal Medicine*.

[22] Ottenbacher KJ, Ottenbacher HR, Tooth L, Ostir GV (2004). A review of two journals found that articles using multivariable logistic regression frequently did not report commonly recommended assumptions. *Journal of Clinical Epidemiology*.

[23] Ducher M, Cerutti C, Marquand A (2005). How to limit screening of patients for atheromatous renal artery stenosis in two-drug resistant hypertension?. *Journal of Nephrology*.

[24] Gevaert O, De Smet F, Kirk E (2006). Predicting the outcome of pregnancies of unknown location: Bayesian networks with expert prior information compared to logistic regression. *Human Reproduction*.

[25] Pozzi C, Bolasco P, Fogazzi G (1999). Corticosteroids in IgA nephropathy: a randomised controlled trial. *The Lancet*.

[26] Roberts ISD, Cook HT, Troyanov S (2009). The Oxford classification of IgA nephropathy: pathology definitions, correlations, and reproducibility. *Kidney International*.

[27] Herzenberg AM, Fogo AB, Reich HN (2011). Validation of the Oxford classification of IgA nephropathy. *Kidney International*.

